# Construction and validation of a nomogram for predicting cancer-specific survival in hepatocellular carcinoma patients

**DOI:** 10.1038/s41598-020-78545-2

**Published:** 2020-12-07

**Authors:** Kang Liu, Gaobo Huang, Pengkang Chang, Wei Zhang, Tao Li, Zhijun Dai, Yi Lv

**Affiliations:** 1 National Local Joint Engineering Research Center for Precision Surgery and Regenerative Medicine, First Affiliated Hospital of Xi’an Jiaotong University, Xi’an, 710061 Shaanxi China; 2 Department of Hepatobiliary Surgery, First Affiliated Hospital of Xi’an Jiaotong University, Xi’an, 710061 Shaanxi China; 3 Department of Breast Surgery, The First Affiliated Hospital, School of Medicine, Zhejiang University, Hangzhou, 310003 Zhejiang China

**Keywords:** Cancer, Oncology

## Abstract

The prognosis of patients with hepatocellular carcinoma (HCC) and intrahepatic cholangiocarcinoma (ICC) is a research hotspot. This study aimed to incorporate important factors obtained from SEER database to construct and validate a nomogram for predicting the cancer-specific survival (CSS) of patients with HCC and ICC. We obtained patient data from SEER database. The nomogram was constructed base on six prognostic factors for predicting CSS rates in HCC patients. The nomogram was validated by concordance index (C-index), the receiver operating characteristic (ROC) curve and calibration curves. A total of 3227 patients diagnosed with HCC (3038) and ICC (189) between 2010 and 2015 were included in this study. The C-index of the nomogram for HCC patients was 0.790 in the training cohort and 0.806 in the validation cohort. The 3- and 5-year AUCs were 0.811 and 0.793 in the training cohort. The calibration plots indicated that there was good agreement between the actual observations and predictions. In conclusion, we constructed and validated a nomogram for predicting the 3- and 5-year CSS in HCC patients. We have confirmed the precise calibration and excellent discrimination power of our nomogram.

## Introduction

Primary liver cancer, the fourth leading cause of cancer-related mortality and the sixth most common cancer, is a global health issue^1,2^. Histologically, primary liver cancer includes three main subtypes: hepatocellular carcinoma (HCC), intrahepatic cholangiocarcinoma (ICC), and mixed hepatocellular cholangiocarcinoma^3^. HCC is the most frequent subtype of liver cancer, which accounts for more than 80% of all primary liver cancers^4,5^, in frequency only to HCC, ICC arises from the epithelial layer of the second-degree biliary tract and is highly malignant^6^. The prognosis of patients with HCC and ICC is a research hotspot because new and more effective treatment strategies need to be based on information regarding prognostic risks. Prognostic factors such as patient age, tumor grade, and the American Joint Committee on Cancer (AJCC) stage have been used to predict patients’ survival time and response to treatment^7,8^. However, oncologists face challenges with the use of these unconsolidated factors; therefore, it is necessary to integrate multiple prognostic factors into an easy-to-use predictive system to better inform oncologists and more accurately stratify patients.

A nomogram is a predictive tool that creates a simple graph based on a predictive statistical model^9^. It can be used to calculate the probability of a clinical event by considering the prognostic weight of each factor. Nomograms have been widely used to assist clinical decision- making^10–12^. This study aimed to incorporate important factors obtained from the Surveillance, Epidemiology, and End Results (SEER) database to construct and validate a nomogram for predicting the cancer-specific survival (CSS) of patients with HCC and ICC.

## Methods

### Ethics statement

Informed patient consent was not required for data obtained from SEER, as cancer is a publicly reportable disease in every state in the USA.

### Study population

We obtained patient data from the SEER 18 Research Data + Hurricane Katrina Impacted Louisiana Cases, Nov 2018 Sub (2010–2015 varying) incidence database, using SEER*Stat version 8.3.6. Patients diagnosed with HCC or ICC (primary site code 220 or 221) were included. Cases that were not confirmed by microscopy or only by autopsy were excluded, as were those with missing or incomplete additional data. The following additional data (variables) were used in the analysis: patient age, race, and sex; AJCC staging for the extent of tumor (T), extent of spread to lymph nodes (N), and presence of metastasis (M); and tumor grade, surgery (Y/N), alpha fetoprotein (AFP) level, fibrosis score, months of survival, and cause of death. We applied the seventh edition of the AJCC staging system, which was available for patients between 2010 and 2015. Ultimately, we identified 3227 eligible patients for inclusion in our study.

### Statistical analysis

For nomogram construction and validation, we randomly divided all the HCC patients into training (n = 2123) and validation (n = 915) cohorts, in a ratio of 7:3^13,14^. Multivariate Cox proportional hazards regression analysis was performed to identify variables (P < 0.05) that significantly affected CSS in the training group. Using these identified prognostic factors, we constructed a nomogram for predicting 3- and 5-year CSS rates in HCC and ICC patients^15^.

The nomogram was validated internally in the training cohort and externally in the validation cohort. To evaluate the discriminative ability of the nomogram, we used the concordance index (C-index) and the receiver operating characteristic (ROC) curve and assessed the area under the curve (AUC)^16,17^. A C-index or AUC of 0.5 indicates a discrimination ability that is no better than chance, whereas that of 1.0 indicates a perfect discrimination ability^18^. Calibration curves were constructed using a bootstrap approach, with 500 resamples, to compare the predicted CSS with the CSS observed in the study.

All statistical analyses were conducted using SPSS (version 24.0; SPSS, Chicago, IL, USA) and R software (version 3.6.1; http://www.r-project.org/). A P value of less than 0.05 was considered to indicate statistical significance.

## Results

### Patient characteristics

A total of 3227 patients diagnosed with HCC (3038) and ICC (189) between 2010 and 2015 were included in this study. The training and validation cohorts of HCC patients consisted of 2123 and 915 cases, respectively, selected by the random split-sample method (split ratio: 7:3). In the total cohort of HCC patients, the majority of patients were under 65 years old (59.4%), white (66.5%), and male (76.0%). Furthermore, most of the patients had T1 (47.6%), N0 (95.7%), and M0 (93.5%); patients with a grade II tumor differentiation degree accounted for 50.5% of all cases. A large proportion of the patients had elevated AFP levels (64.9%) and cirrhosis (69.5%), and 46.6% of patients underwent tumor resection surgery. The characteristics of HCC patients in the training and validation cohorts were similar to those in the total cohort (Table 1).

**Table 1:** HCC patient characteristics in the study.

Characteristics	Total cohort	Training cohort	Validation cohort
	3038 (100%)	2123 (69.9%)	915 (30.1%)
**Age**			
< 65	1804 (59.4%)	1268 (59.7%)	536 (58.6%)
≥ 65	1234 (40.6%)	855 (40.3%)	379 (41.4%)
**Race**			
Black	391 (12.9%)	271 (12.8%)	120 (13.1%)
Other	627 (20.6%)	435 (20.5%)	192 (21.0%)
White	2020 (66.5%)	1417 (66.7%)	603 (65.9%)
**Sex**			
Female	729 (24.0%)	499 (23.5%)	230 (25.1%)
Male	2309 (76.0%)	1624 (76.5%)	685 (74.9%)
**AJCC T**			
T1	1447 (47.6%)	995 (46.9%)	452 (49.4%)
T2	904 (29.8%)	636 (30.0%)	268 (29.3%)
T3	595 (19.6%)	424 (20.0%)	171 (18.7%)
T4	92 (3.0%)	68 (3.2%)	24 (2.6%)
**AJCC N**			
N0	2908 (95.7%)	2025 (95.4%)	883 (96.5%)
N1	130 (4.3%)	98 (4.6%)	32 (3.5%)
**AJCC M**			
M0	2841 (93.5%)	1985 (93.5%)	856 (93.6%)
M1	197 (6.5%)	138 (6.5%)	59 (6.4%)
**Surgery**			
No surgery	1146 (37.7%)	819 (38.6%)	327 (35.7%)
Tumor resection	1415 (46.6%)	968 (45.6%)	447 (48.9%)
Liver transplantation	477 (15.7%)	336 (15.8%)	141 (15.4%)
**Grade**			
I	911 (30.0%)	648 (30.5%)	263 (28.7%)
II	1534 (50.5%)	1055 (49.7%)	479 (52.3%)
III	559 (18.4%)	395 (18.6%)	164 (17.9%)
IV	34 (1.1%)	25 (1.2%)	9 (1.0%)
**AFP**			
Nomal	1065 (35.1%)	749 (35.3%)	316 (34.5%)
Elevated	1973 (64.9%)	1374 (64.7%)	599 (65.5%)
**Fibrosis**			
Normal	928 (30.5%)	662 (31.2%)	266 (29.1%)
Cirrhosis	2110 (69.5%)	1461 (68.8%)	649 (70.9%)

For ICC patients, the training and validation cohorts consisted of 132 and 57 cases respectively. In the total cohort of ICC patients, most of characteristics (Age, Race, Sex, AJCC stage, Surgery and Grade) were similar to HCC patients. On the contrary, the majority of ICC patients had normal AFP levels (73.5%) and fibrosis level (66.7%). The characteristics of ICC patients in the training and validation cohorts were similar to those in the total cohort (Supplementary Table S1).

### Screening for prognostic factors of CSS

For HCC patients, based on univariate and multivariate Cox proportional hazards regression analyses, we identified six independent prognostic factors in the training cohort. AJCC T2/T3/T4 (hazard ratio [HR] = 1.280/2.221/3.352, P < 0.01), AJCC M1 (HR = 2.540, P < 0.001), tumor resection (HR = 0.363, P < 0.001), liver transplantation (HR = 0.096, P < 0.001), grade II/III/IV (HR = 1.234/1.998/2.214, P < 0.05), elevated AFP level (HR = 1.336, P < 0.001) and cirrhosis (HR = 1.356, P < 0.001) were all significantly associated with CSS in HCC patients (Table 2).

**Table 2:** Univariate and multivariate Cox regression analysis based on all variables for HCC patient cancer-specific survival (training cohort).

Characteristics	Univariate analysis		Multivariate analysis	
	HR (95% CI)	P value	HR (95% CI)	P value
**Age**				
< 65	Reference			
≥ 65	1.124 (0.974–1.297)	0.111		
**Race**				
Black	Reference		Reference	
Other	0.742 (0.576–0.955)	0.002*	0.978 (0.755–1.267)	0.87
White	0.916 (0.741–1.133)	0.421	1.080 (0.872–1.336)	0.478
**Sex**				
Female	Reference		Reference	
Male	1.209 (1.02–1.433)	0.029*	1.053 (0.886–1.251)	0.554
**AJCC T**				
T1	Reference		Reference	
T2	1.420 (1.192–1.693)	< 0.001***	1.280 (1.068–1.534)	< 0.01**
T3	3.416 (2.859–4.081)	< 0.001***	2.221 (1.833–2.691)	< 0.001***
T4	6.686 (4.992–8.955)	< 0.001***	3.352 (2.433–4.619)	< 0.001***
**AJCC N**				
N0	Reference		Reference	
N1	3.653 (2.823–4.727)	< 0.001***	0.945 (0.703–1.271)	0.709
**AJCC M**				
M0	Reference		Reference	
M1	5.735 (4.658–7.061)	< 0.001***	2.540 (2.002–3.223)	< 0.001***
**Surgery**				
No surgery	Reference		Reference	
Tumor resection	0.273 (0.235–0.317)	< 0.001***	0.363 (0.307–0.429)	< 0.001***
Liver transplantation	0.074 (0.052–0.107)	< 0.001***	0.096 (0.066–0.139)	< 0.001***
**Grade**				
I	Reference		Reference	
II	1.016 (0.856–1.206)	0.856	1.234 (1.035–1.472)	0.019*
III	2.135 (1.761–2.590)	< 0.001***	1.998 (1.628–2.451)	< 0.001***
IV	2.241 (1.347–3.727)	0.014*	2.124 (1.266–3.565)	< 0.01**
**AFP**				
Nomal	Reference		Reference	
Elevated	1.809 (1.542–2.121)	< 0.001***	1.336 (1.132–1.577)	< 0.001***
**Fibrosis**				
Normal	Reference		Reference	
Cirrhosis	1.196 (1.022–1.398)	0.025*	1.356 (1.150–1.599)	< 0.001***

For ICC patients, only three independent prognostic factors in the training cohort were identified. AJCC T3 + T4 (HR = 1.913, P = 0.015), AJCC M1 (HR = 4.036, P < 0.001), and surgery (HR = 0.328, P < 0.001) were all significantly associated with CSS in ICC patients (Supplementary Table S2).

### Nomogram construction

**Figure 1:**
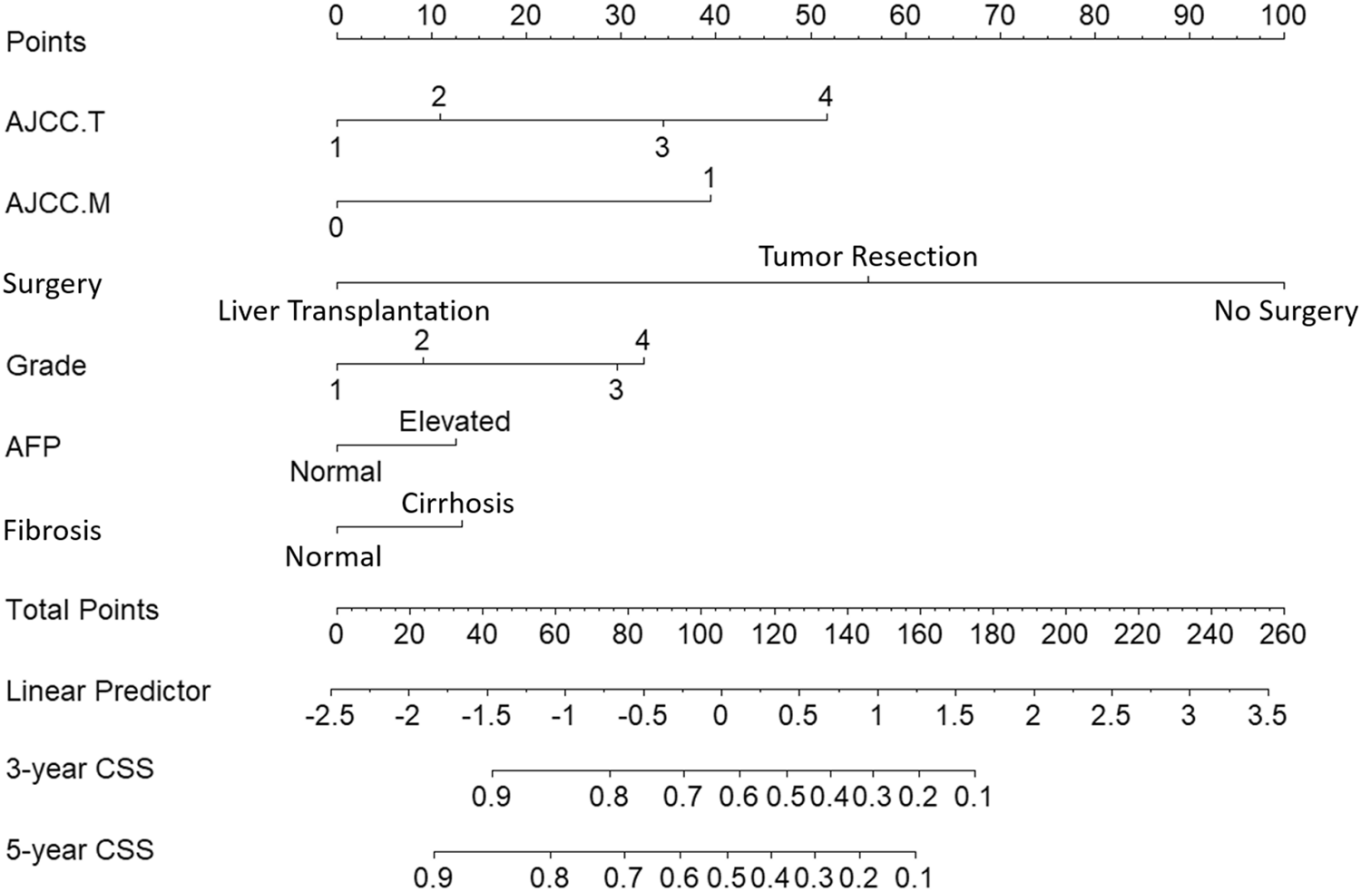
The nomogram predicting CSS in patients with HCC. Each factor was given a point on the basis of the nomograms. The total points were obtained by adding the given points of all factors. The estimated 3- and 5-year probabilities of CSS of the individual patient can be easily obtained from the nomogram based on the total points.

Due to the small sample size (189) and few independent prognostic factors of ICC patients, the nomogram was only constructed for HCC patients. A nomogram based on the selected prognostic factors from the training cohort was developed for the prediction of HCC patient CSS at 3 and 5 years (Fig. 1). The nomogram demonstrated that surgery contributed the most to prognosis, followed by AJCC T, AJCC M, grade, fibrosis and AFP level. Each level of every variable was assigned a score on the points scale. The total score was obtained by adding the scores of each of the selected variables. The prediction corresponding to this total score then helped in estimating the 3- and 5-year CSS for each HCC patient.

### Nomogram validation

**Figure 2:**
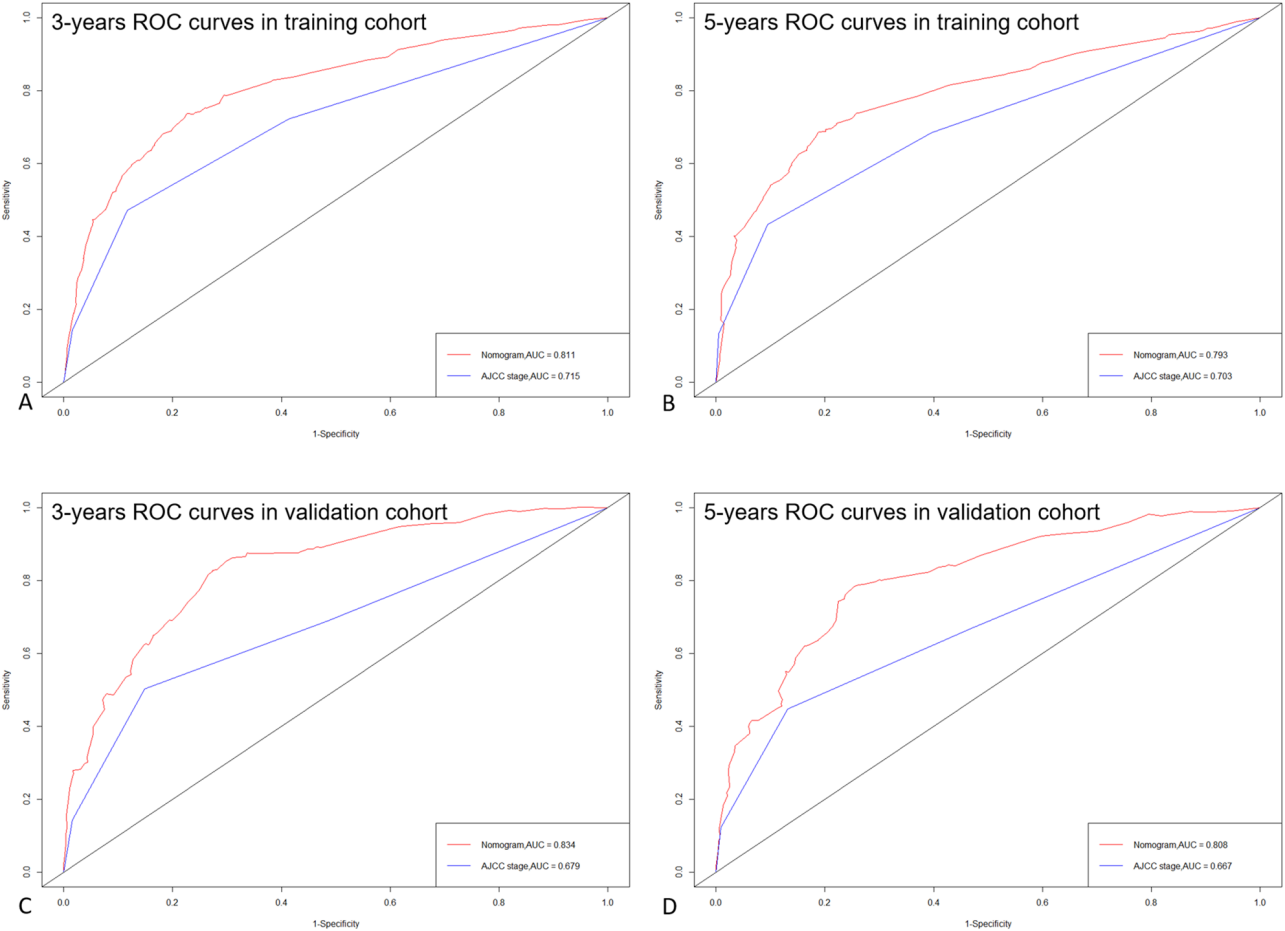
ROC curves of the Nomogram and AJCC stage in prediction of prognosis at 3- (**A**) and 5-year (**B**) point in the training cohort. ROC curves of the Nomogram and AJCC stage in prediction of prognosis at 3- (**C**) and 5-year (**D**) point in the validation cohort.

**Figure 3:**
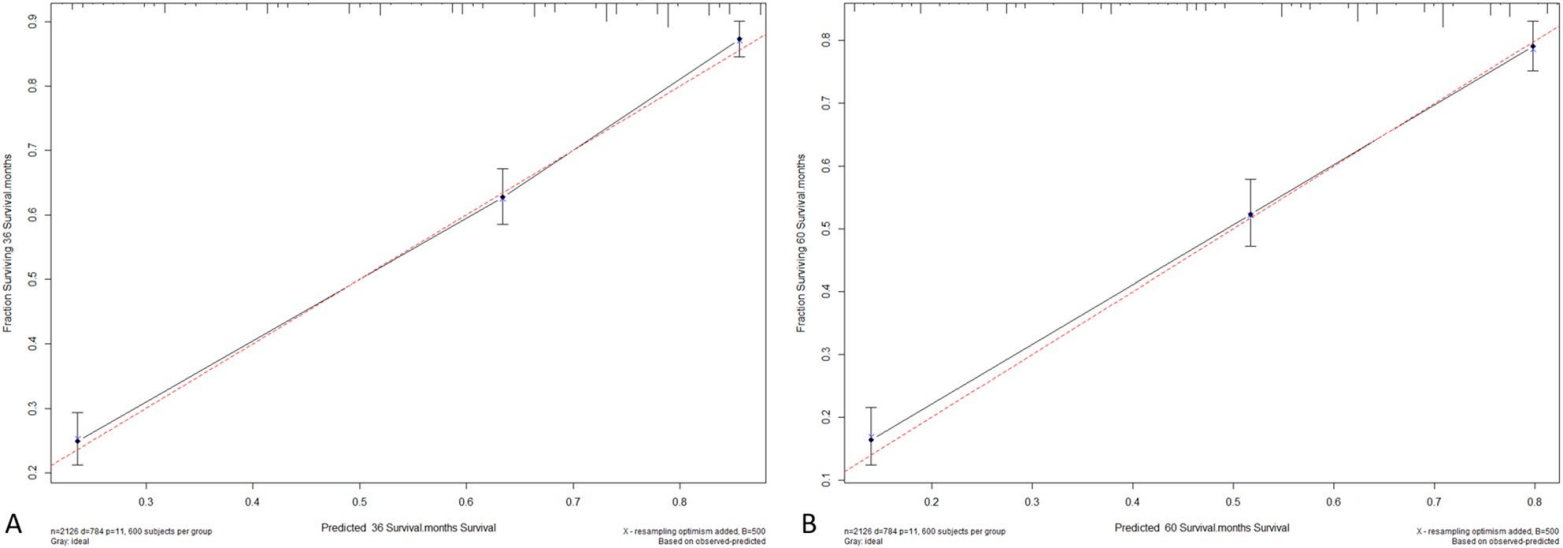
3- (**A**) and 5-years (**B**) calibration curves for probability of HCC patient CSS nomogram construction in training cohort (bootstrap = 500 repetitions).

**Figure 4:**
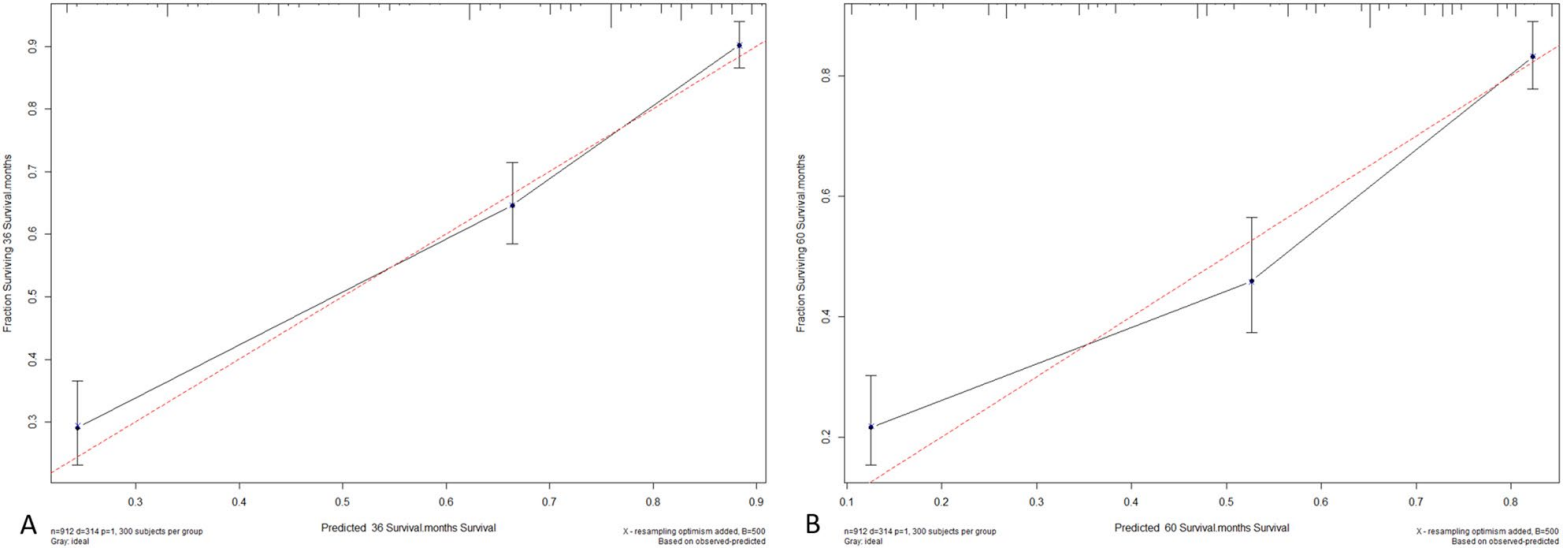
3- (**A**) and 5-years (**B**) calibration curves for probability of HCC patient CSS nomogram construction in validation cohort (bootstrap = 500 repetitions).

The C-index of the nomogram (training cohort = 0.790, validation cohort = 0.806) was higher than which based on the AJCC stage (training cohort = 0.691, validation cohort = 0.675). Furthermore, the AUCs of the nomogram were higher than AJCC stage in both training (3-year AUC: 0.811 vs. 0.715, 5-year AUC: 0.793 vs.0.703, Fig. 2A,B) and validation (3-year AUC: 0.834 vs. 0.679, 5-year AUC: 0.808 vs. 0.667, Fig. 2C,D) cohorts for 3- and 5-year. These results indicate that the discrimination performance of the model was better than traditional AJCC stage in both the training and validation cohorts. The calibration plots for the 3- and 5-year CSS indicated that there was good agreement between the actual observations and predictions made using the nomogram in both the training cohort (Fig. 3) and the validation cohort (Fig. 4).

## Discussion

Determining accurate tumor prognosis after definitive treatment is important^19^. Although the AJCC staging system is widely used for predicting prognosis in primary liver cancer patients, it has inherent defects because it neglects many significant risk factors such as race, age, and grade. Nomograms have been shown to be more accurate and user-friendly than the conventional staging system in many cancers^20,21^. In this study, we constructed a more comprehensive model based on a combination of various risk factors to better predict prognosis in HCC patients.

To identify independent prognostic factors, we performed univariate and multivariate Cox proportional hazards regression analysis. Univariate analysis indicated that race, sex, AJCC T, AJCC N, AJCC M, surgery, grade, AFP level, and fibrosis were potential prognostic factors for HCC patients. After multivariate analysis, only AJCC T, AJCC M, surgery, grade, AFP and fibrosis were independent prognostic factors for HCC patients and the nomogram was constructed based on those six factors. Our results showed that HCC patients who underwent liver transplantation had a better prognosis than those who underwent tumor resection. This is a useful conclusion for both doctors and patients.

For ICC patients, only AJCC T, AJCC M, and surgery were potential prognostic factors after univariate analysis and multivariate analysis. These factors were quite similar to traditional AJCC staging system and the sample size (189) of ICC patients was relatively small. Therefore it was not meaningful to construct a nomogram based on these three factors for ICC patients. A large sample size study including more risk factors in the future would be needed.

It is widely known that a model has relatively good discrimination if its C-index and AUC exceed 0.7^22^; therefore, our model has a good discrimination ability. Furthermore, the calibration plot indicated that the CSS probabilities predicted by our nomogram were identical to the actual ones. The validation results indicate that our nomogram could be effective for application in the clinical setting.

Our study has some limitations. First, this large-sample retrospective study was based on the SEER database, which may have some inherent biases. Second, data regarding several potential important prognosis-related factors such as HBsAg, AST, CEA, and vascular invasion were not available in the SEER database. Third, due to the small sample size (189) and few independent prognostic factors of ICC patients, the nomogram was only constructed for HCC patients. Finally, our nomogram was internally validated, and it needs to be validated externally using other populations.

## Conclusion

We constructed and validated a nomogram for predicting the 3- and 5-year CSS in HCC patients. The proposed nomogram considered six independent risk factors: AJCC T, AJCC M, surgery, tumor grade, AFP level and fibrosis. We have confirmed the precise calibration and excellent discrimination power of our nomogram. The predictive power of this nomogram may be improved by considering other potential important factors that we could not be obtained from the SEER database, and also by external validation.

## Supplementary Information

Supplementary Information

## Data Availability

All data were acquired from the SEER database.
