# Supplementary material for: Construction and validation of a nomogram for predicting cancer-specific survival in hepatocellular carcinoma patients
Source: Sci Rep. 2020 Dec 7;10:21376. doi: 10.1038/s41598-020-78545-2 (PMC7721744; doi:10.1038/s41598-020-78545-2)
Supplement: Supplementary file 1 — Supplementary Information [file 41598_2020_78545_MOESM1_ESM.docx]

Supplementary Table 1. ICC Patient characteristics in the study.

| Characteristics | Total cohort | Training cohort | Validation cohort |
| --- | --- | --- | --- |
|  | 189(100%) | 132(70%) | 57(30%) |
| Age |  |  |  |
| <65 | 106(56.1%) | 77(58.3%) | 29(50.9%) |
| ≥65 | 83(43.9%) | 55(41.7%) | 28(49.1%) |
| Race |  |  |  |
| Black | 14(7.4%) | 11(8.3%) | 3(5.3%) |
| Other | 38(20.1%) | 27(20.5%) | 11(19.3%) |
| White | 137(72.5%) | 94(71.2%) | 43(75.4%) |
| Sex |  |  |  |
| Female | 80(42.3%) | 59(44.7%) | 21(36.8%) |
| Male | 109(57.7%) | 73(55.3%) | 36(63.2%) |
| AJCC T |  |  |  |
| T1 | 81(42.9%) | 52(39.4%) | 29(50.9%) |
| T2 | 44(23.3%) | 33(25.0%) | 11(19.3%) |
| T3 | 46(24.3%) | 31(23.5%) | 15(26.3%) |
| T4 | 18(9.5%) | 16(12.1%) | 2(3.5%) |
| AJCC N |  |  |  |
| N0 | 143(75.7%) | 100(75.8%) | 43(75.4%) |
| N1 | 46(24.3%) | 32(24.2%) | 14(24.6%) |
| AJCC M |  |  |  |
| M0 | 155(82.0%) | 108(81.8%) | 47(82.5%) |
| M1 | 34(18.0%) | 24(18.2%) | 10(17.5%) |
| Surgery |  |  |  |
| No Surgery | 70(37.0%) | 44(33.3%) | 26(45.6%) |
| Tumor Resection | 110(58.2%) | 80(60.6%) | 30(52.6%) |
| Liver Transplantation | 9(4.8%) | 8(6.1%) | 1(1.8%) |
| Grade |  |  |  |
| I | 22(11.6%) | 17(12.9%) | 5(8.8%) |
| II | 90(47.6%) | 60(45.5%) | 30(52.6%) |
| III | 76(40.2%) | 54(40.9%) | 22(38.6%) |
| IV | 1(0.5%) | 1(0.8%) | 0(0.0%) |
| AFP |  |  |  |
| Nomal | 139(73.5%) | 98(74.2%) | 41(71.9%) |
| Elevated | 50(26.5%) | 34(25.8%) | 16(28.1%) |
| Fibrosis |  |  |  |
| Normal | 126(66.7%) | 89(67.4%) | 37(64.9%) |
| Cirrhosis | 63(33.3%) | 43(32.6%) | 20(35.1%) |

Supplementary Table 2. Univariate and multivariate Cox regression analysis based on all variables for ICC patient cancer-specific survival (Training Cohort).

| Characteristics | Univariate analysis | | Multivariate analysis | |
| --- | --- | --- | --- | --- |
|  | HR (95% CI) | P value | HR (95% CI) | P value |
| Age |  |  |  |  |
| <65 | Reference |  |  |  |
| ≥65 | 0.941(0.559-1.584) | 0.821 |  |  |
| Race |  |  |  |  |
| Black | Reference |  |  |  |
| Other | 0.683(0.237-1.969) | 0.481 |  |  |
| White | 0.883(0.350-2.226) | 0.793 |  |  |
| Sex |  |  |  |  |
| Female | Reference |  |  |  |
| Male | 1.314(0.792-2.178) | 0.289 |  |  |
| AJCC T |  |  |  |  |
| T1+T2 | Reference |  | Reference |  |
| T3+T4 | 2.41(1.452-4.001) | **<0.001***** | 1.913(1.129-3.239) | **0.015*** |
| AJCC N |  |  |  |  |
| N0 | Reference |  | Reference |  |
| N1 | 3.606(2.136-6.088) | **<0.001***** | 1.477(0.770-2.831) | 0.24 |
| AJCC M |  |  |  |  |
| M0 | Reference |  | Reference |  |
| M1 | 8.556(4.669-15.68) | **<0.001***** | 4.036(1.911-8.520) | **<0.001***** |
| Surgery |  |  |  |  |
| No Surgery | Reference |  | Reference |  |
| Surgery | 0.199(0.118-0.337) | **<0.001***** | 0.328(0.178-0.604) | **<0.001***** |
| Grade |  |  |  |  |
| I+II | Reference |  | Reference |  |
| III+IV | 1.87(1.139-3.072) | **0.013*** | 1.046(0.602-1.816) | 0.873 |
| AFP |  |  |  |  |
| Nomal | Reference |  |  |  |
| Elevated | 1.455(0.865-2.445) | 0.157 |  |  |
| Fibrosis |  |  |  |  |
| Normal | Reference |  |  |  |
| Cirrhosis | 1.261(0.758-2.098) | 0.372 |  |  |
